# Comparison of transthoracic echocardiography with computed tomography in evaluation of pulmonary veins

**DOI:** 10.1186/s12872-019-01272-8

**Published:** 2019-12-30

**Authors:** Qing-Qing Dong, Wen-Yi Yang, Ya-Ping Sun, Qian Zhang, Guang Chu, Gen-Qing Zhou, Gang Chen, Song-Wen Chen, Shao-Wen Liu, Fang Wang

**Affiliations:** 1 Department of Cardiology, Shanghai General Hospital of Nanjing Medical University, Shanghai, China; 2 Department of Cardiology, Shanghai General Hospital, Shanghai Jiao Tong University School of Medicine, Shanghai, China

**Keywords:** Atrial fibrillation, Catheter ablation, Transthoracic echocardiography, Computed tomography, Pulmonary vein

## Abstract

**Background:**

Transesophageal echocardiography may be used to assess pulmonary veins for atrial fibrillation ablation. No study focused on the role of transthoracic echocardiography (TTE) in evaluating the diameter and anatomy of pulmonary veins.

**Methods:**

Among 142 atrial fibrillation patients (57.7% men; mean age, 60.5) hospitalised for catheter ablation, we assessed pulmonary veins and compared the measurements by TTE with cardiac computed tomography (CT) before ablation. Among 17 patients who had follow-up examinations, the second measurements were also studied.

**Results:**

TTE identified and determined the diameters of 140 (98.6%) right and 140 (98.6%) left superior PVs, and 136 (95.7%) right and 135 (95.1%) left inferior PVs. A separate middle PV ostia was identified in 14 out of the 22 patients (63.6%) for the right side and in 2 out of 4 (50.0%) for the left side. The PV diameters before ablation assessed by CT vs. TTE were 17.96 vs. 18.07 mm for right superior, 15.92 vs. 15.51 mm for right inferior, 18.54 vs. 18.42 mm for left superior, and 15.56 vs. 15.45 mm for left inferior vein. The paired differences between the assessments of CT and TTE were not significant (*P* ≥ 0.31) except for the right inferior vein with a CT-*minus*-TTE difference of 0.41 mm (*P* = 0.018). The follow-up PV diameters by both CT (*P* ≥ 0.069) and TTE (*P* ≥ 0.093) were not different from baseline measurements in the 17 patients who had follow-up measurements.

**Conclusions:**

With a better understanding of PV anatomy in TTE imaging, assessing PV diameters by non-invasive TTE is feasible. However, the clear identification of anatomic variation might still be challenging.

## Background

Atrial fibrillation is one of the most common arrhythmia encountered in clinical practice and associated with increased risk of cardiovascular mortality and morbidity [1, 2], mainly caused by atrial thrombus [3, 4] and irregular tachycardia. Electrical isolation of pulmonary vein (PV) with transcatheter radiofrequency ablation is increasingly applied to treat atrial fibrillation and had a success rate of keeping sinus rhythm ranging from 62 to 90% and reducing cardiovascular morbidity [5, 6]. Accurate assessment on anatomy and variation of the left atrium and PVs is critical to a successful ablation procedure. On the other hand, severe PV stenosis is a major complication after transcatheter ablation, but is commonly ignored [7]. Because of superior spatial resolution and no limitation of acoustic windows, imaging by computed tomography (CT) or magnetic resonance has been widely applied in assessing PV stenosis after ablation [8]. However, cumulative radiation exposure [9], application of nephrotoxic iodine contrast agents during CT scan, claustrophobia during magnetic resonance imaging and the high costs cannot be ignored. Transesophageal echocardiography is an alternative method [10, 11], but still has limitations of the potential risk of oesophageal injury and difficulty for patient tolerance. The non-invasive transthoracic echocardiography (TTE) is routinely performed before and after ablation and might be used to visualise the PVs diameters [12, 13]. The aim of this study was to compare TTE with CT in evaluating anatomy and diameters of PVs before and after catheter ablation for atrial fibrillation.

## Methods

### Study population

Between February 2012 and August 2012, 142 patients had a history of paroxysmal or persistent atrial fibrillation and underwent CT and TTE before atrial fibrillation ablation were enrolled in this study. Patients were excluded if they were younger than 18 years old, or they had hyperthyroidism, congenital heart disease, renal impairment, left atrial appendage thrombus, moderate or severe mitral stenosis or regurgitation, cardiothoracic surgery within 6 months, history of infection within 1 month, or contraindications to oesophageal intubation. All patients underwent clinical examinations, TTE, transesophageal echocardiography, and cardiac CT within 48 h before the ablation procedure. Seventeen of them with recurrent atrial fibrillation had a repeated TTE and cardiac CT at 12 months during the rehospitalisation for a second catheter ablation. The Ethics Committee of Shanghai General Hospital, Shanghai, China, approved the study. All patients gave informed consent.

### Echocardiography

**Fig. 1:**
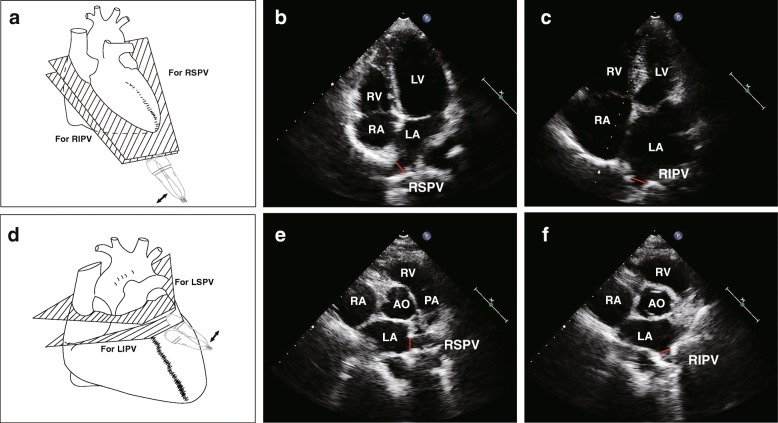
Transthoracic echocardiographic views (**a** and **d**) to detect and measure right superior (RSPV, [**b**]) and inferior (RIPV, [**c**]) and left superior (LSPV, [**e**]) and inferior (LIPV, [**f**]) pulmonary veins. The RSPV and RIPV can be visualised in apical 4-chamber or 5-chamber or between these two classical views (**a**). The LSPV adjoins to the left atrial appendage and the LIPV to descending aorta in the adjusted parasternal short-axis aortic view (**d**). Right and left ventricles (RV and LV) and atria (RA and LA), aorta (AO) and pulmonary artery (PA) are marked as references in the views. The diagrams in panels (**a** and **d**) were drawn by W-Y.Y. and the echocardiographic images in Panels (**b**, **c**, **e** and **f**) were recorded by Q-Q.D.

TTE and transesophageal echocardiography were performed using a Philips iE33 machine (Philips Medical Systems, Eindhoven, Nederland) with a transthoracic (S5–1) and multi-plane transesophageal (S7–2) echocardiographic probes, respectively. TTE included the standard views for measuring left atrium and left ventricular volume and left ventricular ejection fraction using Simpson’s biplane method according to the current guideline [14], and the specific images for assessing pulmonary veins [12, 13]. Colourful and pulsed-wave Doppler imaging was applied as an assistant to localise the PVs. Multiple planes of each view by slight adjustments of the probe were employed to confirm the assessment according to our experience and published methods [12, 13]. In summary, right PVs can be visualised and measured in apical 4-chamber or 5-chamber views or an angle between these two classical views. The inferior right PV is generally perpendicularly jointed to the posterior wall of left atrium, while the superior right PV positions higher and drains into the left atrium (LA) commonly with a tilting angle to the LA posterior-medial wall (Fig. 1). In the parasternal short-axis aortic view, the left superior PV adjoins to the LA appendage angulated to the lateral LA wall. Its inferior counterpart adjoins to descending aorta perpendicular to the posterior wall (Fig. 1). The other views including parasternal long-axis, apical 2-chamber, suprasternal and subcostal were applied to confirm the assessment. In some cases, the extra PV ostia visualised between the superior and inferior veins is identified as the middle PV. For ruling out thrombus in the left atrium or left atrial appendage for the ablation procedure, we routinely performed transesophageal echocardiography after TTE under conscious sedation and oral lidocaine.

Echocardiographic values were average of three consecutive measurements over 2-s digital loops for patients with atrial fibrillation rhythm and three-beat loops for patients in normal sinus rhythm. The PV diameters were determined at the site of around 5 mm from ostia during left ventricular end-systole. All echocardiographic images were obtained and offline analysed by 2 experienced cardiologists (Y-P.S. and Q-Q.D.) who were blind to the results of cardiac CT. In a subset of 30 patients, the agreements assessed as intraclass coefficient correlation for the four-PV measurements between the two echocardiographers ranged from 0.84 to 0.91 (*P* < 0.001). The Kappa statistics assessing the intra-observer agreement for detecting any PV variation among these 30 patients was 0.53 (95% CI, 0.08–0.97; *P* = 0.001).

### Computed tomography

We conducted CT scans by using a GE Lightspeed 64 scanner (General Electric Healthcare China, Beijing, China) without heart beat-lowering medication. According to a routine protocol for imaging the PVs, the tube voltage and current were set as 120 kV and 100 mA with a temporal resolution in the range of 185 to 210 ms and a slice thickness of 1.5 mm keeping a 1-mm overlap. Field of view was set to 40 cm. An amount of 100 to 120 mL contrast material (Lopamiro®370 mg I/ml, Shanghai Bracco Sine Pharmaceutical Corp Ltd) was injected via ulnar vein approach at a flow rate of 3.0 to 3.5 mL/s. All raw data of axial imaging were automatically transferred to post-processing workstation (GE Advantage workstation 4.3). By using the multiplanar reconstruction technique, we aimed to assess the maximum diameter in the root of each PV. A cardiologist (G.C.1) who was experienced in cardiac radiology and blinded to the echocardiography results reviewed the post-processed images and measured the maximal diameter of elliptical PVs at the site of around 5 mm from ostia.

### Other measurements and catheter procedures

Medical records were reviewed to obtain the medical history and diagnosis of disease and the laboratory results of serum glucose and creatinine. The detailed ablation protocol was previously published [15, 16]. The ablation technique involves radiofrequency isolation of the PV antrum through a transseptal route guided by CARTO mapping (Biosense Webster, Los Angeles, USA). In some proper cases, the superior vena cava and/or coronary sinus were additionally ablated. All procedures were performed by experienced electrophysiologists (S.-W.L., G.C.2 and S.-W.C.) under heparin anticoagulation according to current guideline [17].

### Statistical analysis

For statistical analysis, we used SAS software version 9.4 (Cary, North California, USA). All categorical variables were summarised as proportions and continuous variables as mean ± SD, except for the duration of atrial fibrillation, which was reported as geometric means and interquartile range. The duration of atrial fibrillation needed a logarithmic transformation for normal distribution. Paired Student’s t-test and z-test were used for comparisons of continuous variables if proper. Fisher’s exact test was applied for comparisons of categorical variables. The Kappa statistic was used to assess the agreement between TTE and CT in detecting PV anatomic variation. To assess the agreement between CT and TTE measurements of PV diameters, intra-class coefficient correlation and limits of agreement by Bland-Altman approach were computed. Finally, Bland-Altman plotted the PV diameter differences over averaged values of CT and TTE measurements per each individual. Significance was a two-tailed α-level of 0.05 or less.

## Results

### Characteristics of participants

Table 1 lists the characteristics of the subjects with (*n* = 17) and without (*n* = 125) a follow-up examination. All 142 patients aged 60.5 ± 10.1 years underwent TTE and cardiac CT on the same day before PV ablation. Among them, 82 (57.7%) were men and 5 (3.5%) had previous ischemic stroke. The duration of atrial fibrillation was 31.8 months (interquartile range, 12–84). Gender, smoking status, history of hypertension, diabetes, heart failure and age, body mass index and estimated glomerular filtration rate and duration of atrial fibrillation were not different (*P* ≥ 0.084) between the patients with and without a follow-up examination. Compared with those 125 subjects who did not have the second examination, 17 participants with the follow-up assessment at 12 months (347–372 days) had an increased heart rate (92.5 vs. 81.7 beats/min, *P* = 0.044) and a higher prevalence of persistent atrial fibrillation (64.7% vs. 24.0%, *P* = 0.0012) before the first ablation.

**Table 1:** Characteristics of the participants at first imaging examination

Characteristic	Patients without/with follow-up examination		All	*P*-value
	Without	With		
N° in category	125	17	142	
N° of participants (%)				
Age ≥ 75 years old	8 (6.4)	1 (5.9)	9 (6.3)	0.99
Men	72 (57.6)	10 (58.8)	82 (57.7)	0.99
Current smoker	16 (12.8)	1 (5.9)	17 (12.0)	0.70
Hypertension	68 (54.4)	8 (47.1)	76 (53.5)	0.61
Diabetes mellitus	11 (8.8)	4 (23.5)	15 (10.6)	0.084
Coronary artery disease	10 (8.0)	0 (0)	10 (7.0)	0.61
Congestive heart failure	8 (6.4)	1 (5.9)	9 (6.3)	0.99
Stroke	4 (3.2)	1 (5.9)	5 (3.5)	0.48
Mean (±SD) of characteristic				
Age	60.5 ± 10.1	60.5 ± 10.1	60.5 ± 10.1	0.99
Body mass index, kg/m^2^	24.2 ± 2.0	24.9 ± 3.2	24.2 ± 2.2	0.16
Heart rate, beats/minute	81.7 ± 20.3	92.5 ± 21.5	83.0 ± 20.6	0.044
eGFR, mL/min/1.73m^2^	89.7 ± 13.3	88.7 ± 16.8	93.5 ± 9.1	0.78
Atrial fibrillation				
Duration of AF, month	31.6 (12–84)	34.3 (12–96)	31.8 (12–84)	0.79
Paroxysmal AF	79 (63.2)	5 (29.4)	84 (59.2)	0.016
Persistent AF	30 (24.0)	11 (64.7)	41 (28.9)	0.0012
Atrial flutter	10 (8.0)	1 (5.9)	11 (7.8)	0.99
Atrial tachycardia	6 (4.8)	0 (0)	6 (4.2)	0.99

Hypertension was a blood pressure of ≥140 mmHg systolic or ≥ 90 mmHg diastolic or use of antihypertensive drugs. Diabetes mellitus was a fasting plasma glucose level of ≥7.0 mmol/L or use of antidiabetic agents. The values were geometric means (interquartile ranges) for TSH and duration of atrial fibrillation, which needed a logarithmic transformation for normal distribution. Estimated glomerular filtration rate (eGFR) was calculated by the CKD-EPI creatinine Eq. (2009). The *P* values are for the significance of the differences between participants with and without follow-up examination

*AF* atrial fibrillation

Compared with those 125 subjects did not have a second ablation (Table 2), the 17 patients went through a repeated ablation procedure had larger left atrial diameter (46.2 vs. 41.6), left ventricular end-diastolic (51.2 vs. 48.8) and systolic (34.1 vs. 32.0) dimensions at baseline (*P* ≤ 0.048). Among these 17 patients, the second measurements of left atrial diameter (42.3 vs. 46.2) and left ventricular end-diastolic dimension (49.4 vs. 51.2) significantly decreased (*P* ≤ 0.041). The changes of left ventricular end-systolic dimension and ejection fraction did not reach significance (*P* ≥ 0.29).

**Table 2:** Comparisons of conventional echocardiographic left atrial and ventricular measurements

Characteristic	Patients without/with follow-up examination			Baseline vs. Follow-up	
	Without	with	*P*-value	Follow-up	*P*-value
N° in category	125	17		17	
LA diameter, mm	41.6 ± 6.4	46.2 ± 5.7	0.0067	42.3 ± 5.5	0.00059
LV end-diastolic dimension, mm	48.8 ± 4.1	51.2 ± 4.5	0.026	49.4 ± 5.6	0.041
LV end-systolic dimension, mm	32.0 ± 4.1	34.1 ± 4.4	0.048	32.9 ± 5.7	0.29
LV ejection fraction, %	63.1 ± 6.3	60.5 ± 6.7	0.12	60.9 ± 8.8	0.89

*P* values are for differences between participants with baseline and follow-up or for paired differences of measurements at baseline and follow-up. LA diameter was assessed from parasternal view. Baseline and follow-up assessments refer to the measurements before PV ablation and at 12 months after ablation, respectively

*LA* left atrial, *LV* left ventricular

### Anatomic variations

Among 142 patients, 618 PVs (331 right plus 287 left) were detected during a cardiac CT scan. Twenty-two patients had one (*n* = 20, 14.1%) or two (*n* = 2, 1.4%) right middle PVs. Three patients had two (*n* = 2, 1.4%) or three (*n* = 1, 0.7%) separate ostia of right superior PV to left atrium. For the right inferior PV, 2 (1.4%) patients had two ostia, 2 (1.4%) patients had three, and 1 (0.7%) patient had four. Left middle PV were observed in 4 patients (2.8%), and the common left PV with joined left superior and inferior PVs to antrum was found in 1 (0.7%) patient. There were 4 patients who had more than 3 right PVs.

No matter with or without additional variations, 140 (98.6%) of right and 140 (98.6%) of left superior PVs, 136 (95.7%) right and 135 (95.1%) left inferior PVs were identified, evaluated and measured for the diameter by TTE. The rest superior or inferior PVs were not visualised because of insufficient acoustic windows or variations in 10 patients. As a variation, the separate middle PV ostia was identified by TTE in 14 out of the 22 patients (63.6%) for the right side and in 2 out of 4 (50.0%) for the left side. Other anatomic variants cannot be clearly identified by using TTE. The Kappa statistic assessing the agreement between TTE and CT in detecting any PV variation was 0.47 (95% CI, 0.29–0.65; *P* < 0.001).

### PV diameter measured by CT vs. TTE at baseline and follow-up

**Fig. 2:**
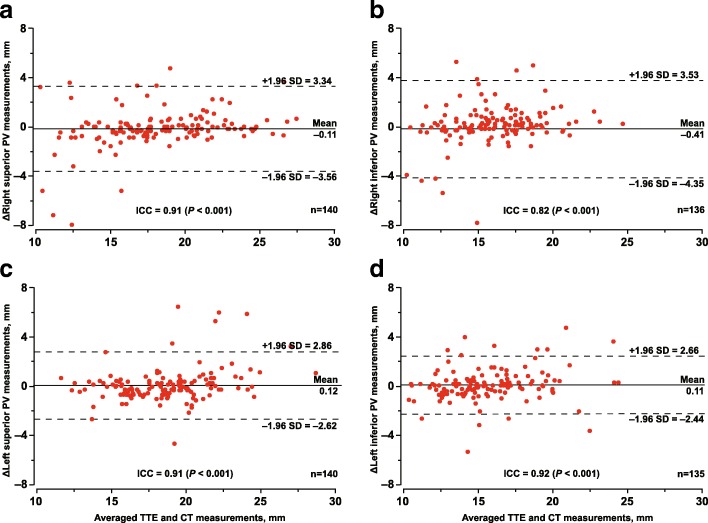
Bland-Altman plots illustrating the diameter differences (CT–TTE) of right superior (**a**), right inferior (**b**), left superior (**c**) and left inferior (**d**) pulmonary veins (PV) assessed by computed tomography (CT) and transthoracic echocardiography (TTE). ICC refers to intraclass coefficient correlation. The 95% limits of agreement are presented as ±1.96 SD

On cardiac CT, PVs and their ostia were generally elliptical in morphology. No matter measured by CT or TTE (Table 3), the superior PVs were bigger than their inferior counterparts (*P* < 0.001). As showed in Tables 3 and 4, the PV diameters before ablation assessed by CT vs. TTE were 17.96 vs. 18.07 mm for the right superior, 15.92 vs. 15.51 mm for the right inferior, 18.54 vs. 18.42 mm for the left superior, and 15.56 vs. 15.45 mm for the left inferior vein. The paired differences between CT and TTE assessments were not significant (*P* ≥ 0.31) except for the right inferior vein with a CT-*minus*-TTE difference of 0.41 mm (*P* = 0.018). The sensitivity analysis in 109 patients without PV anatomic variant was confirmatory (Table 5). The intraclass coefficient correlation (≥ 0.82) and limits of agreement by Bland-Altman method showed a good agreement between CT and TTE measurements (Fig. 2). However, the paired difference of the individual subjects (Fig. 2) ranged − 7.9 to 4.8 mm for right superior, − 7.8 to 5.0 mm for right inferior, − 4.6 to 6.5 mm for left superior, and − 5.3 to 4.7 mm for left inferior PVs.

**Table 3:** Comparison of ostial diameters of the superior vs. inferior pulmonary veins by CT and TTE at baseline

Characteristic	CT measurements			TTE measurements		
	N°	Diameter (mm)	*P*-value	N°	Diameter (mm)	*P*-value
All right PVs						
Superior	142	18.03 ± 4.32		140	18.07 ± 3.79	
Inferior	142	15.94 ± 3.30		136	15.97 ± 2.79	
Paired right PVs						
Superior	142	18.03 ± 4.32		134	17.98 ± 3.69	
Inferior	142	15.94 ± 3.30		134	15.92 ± 2.76	
Difference	142	2.09 ± 4.62	< 0.001	134	2.04 ± 3.98	< 0.001
All left PVs						
Superior	141	18.57 ± 3.42		140	18.42 ± 3.01	
Inferior	141	15.64 ± 3.23		135	15.45 ± 3.01	
Paired left PVs						
Superior	141	18.57 ± 3.42		132	18.47 ± 3.05	
Inferior	141	15.64 ± 3.23		132	15.40 ± 2.93	
Difference	141	2.90 ± 3.89	< 0.001	132	3.06 ± 3.47	< 0.001

*P* values are for paired superior-minus-inferior PV differences of ostial diameters by CT or TTE. There was a patient with a common left PV joined the superior and inferior branches. All anatomically aberrant PVs were excluded for the comparisons. For TTE, only those cases with measurements for both superior and inferior PVs were taken into account for the paired comparison

*PV* pulmonary vein, *CT* computed tomography, *TTE* transthoracic echocardiography

**Table 4:** Measurements of the ostial diameter of pulmonary veins by CT and TTE

Characteristic	Baseline			Follow-up			Baseline minus follow-up		
	N°	Diameter (mm)	*P*-value	N°	Diameter (mm)	*P*-value	N°	Diameter (mm)	*P*-value
Right superior PV									
CT	140	17.96 ± 4.30		17	19.81 ± 4.58		17	0.78 ± 3.72	0.40
TTE	140	18.07 ± 3.79		17	19.21 ± 4.04		17	1.17 ± 3.44	0.18
CT *minus* TTE	140	−0.11 ± 1.76	0.45	17	0.60 ± 0.75	0.0046			
Right inferior PV									
CT	136	15.92 ± 3.19		15	16.76 ± 2.75		15	−0.07 ± 3.52	0.94
TTE	136	15.51 ± 2.90		15	15.37 ± 3.06		15	0.71 ± 4.00	0.50
CT *minus* TTE	136	0.41 ± 2.01	0.018	15	1.39 ± 1.59	0.0044			
Left superior PV									
CT	140	18.54 ± 3.44		17	19.75 ± 3.37		17	0.39 ± 3.16	0.62
TTE	140	18.42 ± 3.01		17	19.54 ± 3.12		17	0.39 ± 2.72	0.56
CT *minus* TTE	140	0.12 ± 1.40	0.33	17	0.21 ± 1.10	0.45			
Left inferior PV									
CT	134	15.56 ± 3.25		14	15.14 ± 3.73		14	2.28 ± 4.18	0.062
TTE	134	15.45 ± 3.01		14	14.89 ± 3.32		14	1.67 ± 3.45	0.093
CT *minus* TTE	134	0.11 ± 1.30	0.31	14	0.25 ± 0.61	0.15			

*P* values are for paired differences of PV ostial diameters by CT vs. TTE or between baseline and follow-up measurements. Baseline and follow-up diameters refer to the measurements before PV ablation and at 12 months after ablation, respectively. Anatomically aberrant PVs were not taken into account for these comparisons

*PV* pulmonary vein, *CT* computed tomography, *TTE* transthoracic echocardiography

**Table 5:** Baseline measurements of the ostial diameter of pulmonary veins by CT and TTE in 109 patients without anatomic aberrance

Characteristic	CT		TTE		CT minus TTE	
	N°	Diameter (mm)	N°	Diameter (mm)	Difference (mm)	*P*-value
Right superior PV						
All	109	18.97 ± 3.86	107	18.72 ± 3.56		
Paired	107	18.90 ± 3.85	107	18.72 ± 3.56	0.18 ± 1.27	0.15
Right inferior PV						
All	109	16.42 ± 3.07	104	15.77 ± 2.88		
Paired	104	16.40 ± 2.89	104	15.77 ± 2.88	0.64 ± 1.44	< 0.001
Left superior PV						
All	109	18.64 ± 3.55	107	18.46 ± 3.08		
Paired	107	18.61 ± 3.57	107	18.46 ± 3.08	0.15 ± 1.35	0.27
Left inferior PV						
All	109	15.73 ± 3.34	104	15.53 ± 3.10		
Paired	104	15.64 ± 3.35	104	15.53 ± 3.10	0.11 ± 1.24	0.37

*P* values are for paired differences of PV ostial diameters measured by CT vs. TTE. The 33 patients with any anatomic aberrance in PVs detected by CT scan were all excluded from the analysis

*PV* pulmonary vein, *CT* computed tomography, *TTE* transthoracic echocardiography

During the follow-up (Table 4), the diameters determined by CT vs. TTE were 19.81 vs. 19.21 for right superior (*P* = 0.0046), 16.76 vs. 15.37 for right inferior (*P* = 0.0044), 19.75 vs. 19.54 for left superior (*P* = 0.45), and 15.14 vs. 14.89 for left inferior vein (*P* = 0.15). Compared with baseline data, the follow-up PV diameters by either CT (*P* ≥ 0.062) or TTE (*P* ≥ 0.093) were not different.

## Discussion

**Fig. 3:**
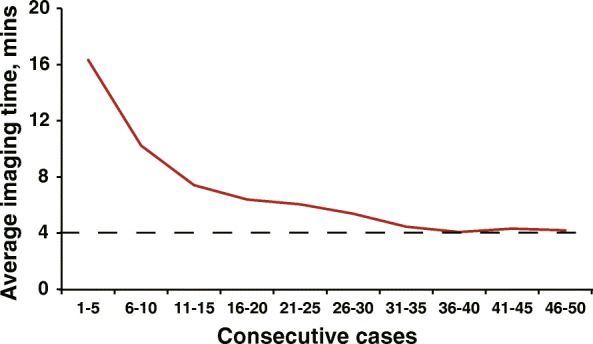
Learning curve of obtaining transthoracic echocardiographic images of pulmonary veins (PV). The X axis refers to the first 50 consecutive study patients who had been imaged by one of the echocardiographer (Q-Q.D.). The Y axis presents the average time spent on obtaining the optimal views to measure PVs per five cases

The current study is the first observational study to compare the TTE with cardiac CT in assessing of PV anatomic variation and diameters. The key finding was that TTE identified 96.8% PVs and produced comparable measurements of PV diameters to cardiac CT scan before and after PV ablation with a limitation to illustrate the detailed information about PV anatomic variations. The low Kappa statistic assessing the agreement between TTE and CT in detecting PV variation was mainly driven by the 10 middle left or right PVs (7.0%) or other rare variations that were not identified by TTE. In the majority of the patients, with or without variations, 96.8% of overall PVs can be detected and evaluated by TTE. As illustrated in Fig. 3, an experienced echocardiographer can obtain optimal TTE images for assessing PVs in about 4 min per patient after 35 cases of performance. Our study showed that TTE can be used to evaluate PVs before ablation, and can be considered as a potential routine follow-up approach for detecting PV stenosis after ablation.

Cardiac CT scan, with a high spatial resolution and no limitation of acoustic windows, is the golden standard tool in assessing PV diameters, morphology and anatomy. The cardiac CT is routinely applied before ablation for atrial fibrillation [17] and can provide accurate diagnosis of the pulmonary vein stenosis [8]. However, cumulative radiation exposure [9] and application of nephrotoxic iodine contrast agents limit the routine use of cardiac CT examination for assessing pulmonary vein stenosis after ablation. As an highly recommended examination during the pre-ablation period to rule out the presence of thrombus in the left atrium and its appendage [17], transesophageal echocardiography can effectively visualise PV diameters, anatomy and the dynamic evaluation of blood flow characteristics in PVs, and therefore provides very important information to identify PV stenosis [10, 11]. Recently, the Role of Transesophageal Echocardiograph Compared to Computed Tomography in Evaluation of Pulmonary Vein Ablation for Atrial Fibrillation (ROTEA) study focused on the comparison of the diameter assessment by transesophageal echocardiography with cardiac CT [11]. Among 43 patients with a mean age of 56 years, To and the coworkers reported that transesophageal echocardiography identified 98% of PVs with obtained adequate Doppler measurement in the pre-ablation study [11]. However, in the studies [18, 19] of 3D transesophageal echocardiography-guided ablation, the feasibility of assessing PV ostia by emerging 3D transesophageal echocardiography was still not confirmatory. In addition, the invasive nature, low tolerance in some patients and risk of oesophageal injury preclude its use as a routine examination after ablation [20]. As far as we know, there was no study compared PV assessment by TTE with cardiac CT scan, invasive angiography or magnetic resonance imaging.

Given the fact that PVs locate far from the probe in the TTE echo window, and the subjective opinion described in the literature and textbooks, the role of TTE on assessing PV anatomy and diameter was usually misunderstood [12, 13]. Conventionally, only right superior or inferior PV is commonly analysed to determine PV flow velocity, mainly for assistant diagnosis of left ventricular diastolic function and mitral regurgitation. By using cardiac catheterisation angiography as a reference, Huang et al. [12] studied 20 adult patients with atrial septal defect hospitalised for interventional closure and described the detail methods to visualise the four PVs. Recently, Gölbaşı and colleagues reported their experience of visualisation of four PVs by using TTE [13]. Based on our experience and these published methods [12, 13], we studied for the first time the role of TTE on analysing PV anatomy and diameter, compared with cardiac CT angiography. In the current study, superior PVs were general bigger in size than its inferior counterparts and 96.8% PVs were visualised and measured by TTE. The PV diameters measured by TTE at baseline and during follow-up were comparable to the CT measurements in the cross-sectional analyses. However, the clear identification of anatomic variation, including existing middle PV and separate ostia of superior and inferior PVs to left atrium might still be challenging in some cases. By using the aforementioned approaches and according to drainage angulation and adjoined anatomic structures, in the majority of the patients, it was not difficult to distinguish between superior and inferior PVs or to identify the superior or inferior PVs even when its counterpart at the same side was not detected. Moreover, confirmatory results of the sensitivity analysis by excluding the patients with any anatomic aberrance detected by cardiac CT imaging indicated that the main findings of the current study were not likely driven by mis-distinguishing the superior, middle or inferior PVs.

The severe PV stenosis (diameter reduction ≥75%) has been considered as a major complication after ablation treatment for atrial fibrillation with life-threatening prognosis if not identified and treated properly [17]. However, in terms of the unspecific clinical manifestation, no guideline-based consensus on the routine follow-up screening of PV stenosis since lack of non-invasive examination methods [17], PV stenosis is usually misdiagnosed. In a previous study, PV diameters significantly decreased after ablation by 0.20 and 0.22 cm similarly as measured by CT and transesophageal echocardiography, respectively, although transesophageal echocardiography underestimated PV ostial diameters for 0.16 to 0.43 cm (*p* < 0.001) compared with CT except for right superior PV (*P* = 0.284) [11]. However, in the present study, in 17 cases who had both CT scan and TTE during follow-up, the longitudinal analyses found that there were no changes in PV diameters by both CT (*P* ≥ 0.069) and TTE (*P* ≥ 0.093) imaging. Indeed, the ablation was mainly performed in pulmonary antra, not inside pulmonary veins, the PV stenosis decreased from 42% [21] two decades ago to less than 1% around 2010 [22]. However, the rate of severe PV stenosis during 12-month follow-up increased again to 3.1% when the new technology of cyroablation was applied [23]. A non-invasive approach to screen PV stenosis after ablation is still in high demand for daily clinical practice. Although the current follow-up analysis only included 17 patients, the baseline comparison with CT scan can also be reasonably extrapolated for the follow-up examination. Of course, a large prospective study focusing on the follow-up changes of PV diameters by TTE will be warranted.

Our studies should be interpreted with limitations. First, a small number of patients had a follow-up examination. However, the clinical characteristics of those patients were similar to those without a follow-up study, except for the heart rate and proportions of persistent and paroxysmal atrial fibrillation. Second, our follow-up comparison only included patients with recurrent atrial fibrillation. Recovery of sinus rhythm after ablation may change the haemodynamics and drainage of left atrium and PV to enhance flow volume and velocity [24, 25]. Third, the angulation of PV flow to ultrasonic beam especially when analysing left PVs, precluded the study of PV flow velocity as commonly conducted in transesophageal studies. Finally, the current study only included data from a single centre. The replication from other studies is needed to confirm our findings.

## Conclusions

TTE was able to identify 96.8% PVs and yielded the comparable measurements of PV diameters to cardiac CT scan before and after PV ablation. As a non-invasive approach compared with CT and TEE, no x-ray exposure and less time-consuming, transthoracic echocardiography is potentially a routine tool for assisting in PVs evaluation before ablation and for screening stenosis of pulmonary veins after ablation when CT or TEE is not generally indicated. However, caution should be taken to check the PV anatomic variation. On the other hand, larger prospective studies are needed to validate our findings.

## Data Availability

The datasets used and/or analysed during the current study are available from the corresponding author on reasonable request.

## Abbreviations

AO: Aorta
CT: Computed tomography
LA: Left atrium
LI: Left inferior
LS: Left superior
LV: Left ventricle
PA: Pulmonary artery
PV: Pulmonary vein
RA: Right atrium
RI: Right inferior
RL: Right superior
RV: Right ventricle
TTE: Transthoracic echocardiography
